# Clinical translation of ^18^F-fluoropivalate – a PET tracer for imaging short-chain fatty acid metabolism: safety, biodistribution, and dosimetry in fed and fasted healthy volunteers

**DOI:** 10.1007/s00259-020-04724-y

**Published:** 2020-03-02

**Authors:** Suraiya R. Dubash, Nicholas Keat, Kasia Kozlowski, Chris Barnes, Louis Allott, Diana Brickute, Sam Hill, Mickael Huiban, Tara D. Barwick, Laura Kenny, Eric O. Aboagye

**Affiliations:** 1 Department of Surgery and Cancer, Imperial College London, GN1 Commonwealth Building, Hammersmith Hospital, DuCane Rd, London, W12 0NN UK; 2 Department of Oncology, Imperial College Healthcare NHS Trust, London, UK; 3 Invicro Ltd, London, UK; 4 Department of Radiology/Nuclear Medicine, Imperial College Healthcare NHS Trust, London, UK

**Keywords:** ^18^F-Fluoropivalate, ^18^F-FPIA, Positron emission tomography, Short chain fatty acid metabolism, Dosimetry, Carnitine

## Abstract

**Background:**

Fatty acids derived de novo or taken up from the extracellular space are an essential source of nutrient for cell growth and proliferation. Radiopharmaceuticals including ^11^C-acetate, and ^18^F-FAC (2-^18^F-fluoroacetate), have previously been used to study short-chain fatty acid (SCFA) metabolism. We developed ^18^F-fluoropivalate (^18^F-FPIA; 3-^18^F-fluoro-2,2-dimethylpropionic acid) bearing a *gem*-dimethyl substituent to assert metabolic stability for studying SCFA metabolism. We report the safety, biodistribution, and internal radiation dosimetry profile of ^18^F-FPIA in 24 healthy volunteers and the effect of dietary conditions.

**Materials and methods:**

Healthy volunteer male and female subjects were enrolled (*n* = 24), and grouped into 12 fed and 12 fasted. Non-esterified fatty acids (NEFA) and carnitine blood measurements were assessed. Subjects received 159.48 MBq (range, 47.31–164.66 MBq) of ^18^F-FPIA. Radiochemical purity was > 99%. Safety data were obtained during and 24 h after radiotracer administration. Subjects underwent detailed multiple whole-body PET/CT scanning with sampling of venous bloods for radioactivity and radioactive metabolite quantification. Regions of interest were defined to derive individual and mean organ residence times; effective dose was calculated using OLINDA 1.1.

**Results:**

All subjects tolerated ^18^F-FPIA with no adverse events. Over 90% of radiotracer was present in plasma at 60 min post-injection. The organs receiving highest absorbed dose (in mGy/MBq) were the liver (0.070 ± 0.023), kidneys (0.043 ± 0.013), gallbladder wall (0.026 ± 0.003), and urinary bladder (0.021 ± 0.004); otherwise there was low tissue uptake. The calculated effective dose using mean organ residence times over all 24 subjects was 0.0154 mSv/MBq (SD ± 0.0010). No differences in biodistribution or dosimetry were seen in fed and fasted subjects, though systemic NEFA and carnitine levels reflected fasted and fed states.

**Conclusion:**

The favourable safety, imaging, and dosimetric profile makes ^18^F-FPIA a promising candidate radiotracer for tracing SCFA metabolism.

**Electronic supplementary material:**

The online version of this article (10.1007/s00259-020-04724-y) contains supplementary material, which is available to authorized users.

## Introduction

^18^F-Fluorodeoxyglucose (^18^F-FDG) positron emission tomography (PET), initially developed to permit elucidation of the early steps of glycolysis, is used clinically as a non-invasive imaging surrogate of glucose metabolism in patients, with applications ranging from detection of cancer to assessment of neurodegeneration, cardiovascular function, inflammation, and infection. Fatty acid metabolism, however, has been less studied by non-invasive molecular imaging.

Glucose and fatty acids dominate as energy sources required for normal cellular homeostasis. Different tissues in the body including the cardiac, liver, skeletal muscle, and adipose tissue exhibit different degrees of “metabolic plasticity” involving reciprocal regulation of glucose and fatty acid metabolism [1]. Fatty acids originate from 3 main sources: exogenous fatty acids from the blood or gastrointestinal tract, de novo synthesis from acetyl-CoA, and the hydrolysis of acylated proteins, phospholipids, and trigylcerides [2]. In addition to aberrant increased glucose utilisation of tumour cells, also known as the Warburg effect [3], de novo fatty acid metabolism is recognised to play a pivotal role in membrane synthesis and proliferation of cancer cells [4]. To obtain energy, fatty acids are broken down by the process of β-oxidation in the mitochondria in eukaryotes. This cyclical series of reactions generates acetyl-CoA, which enters the citric acid cycle, and nicotinamide adenine dinucleotide (NADH) and flavin adenine dinucleotide (FADH2), which are co-enzymes used in the electron transport chain to produce ATP [5]. Short-chain carboxylates including acetate, propionate, butyrate, and the non-natural substrate pivalate (trimethylacetate) use the early steps of the fatty acid oxidation pathway involving acyl-CoA and acyl-carnitine synthesis. However, unlike acetate, pivalate cannot be oxidized to carbon dioxide in mammalian cells as it bears a *tert-*butyl substituent [6]. Pivalate is esterified in vivo in normal tissue to pivaoloylcarnitine (3-[(2,2-dimethylpropanoyl)oxy]-4-(trimethylazaniumyl)butanoate); the resulting ester enters plasma and is either taken up by cells in an L-carnitine-inhibited manner or rapidly eliminated in urine [7–9].

**Fig. 1:**
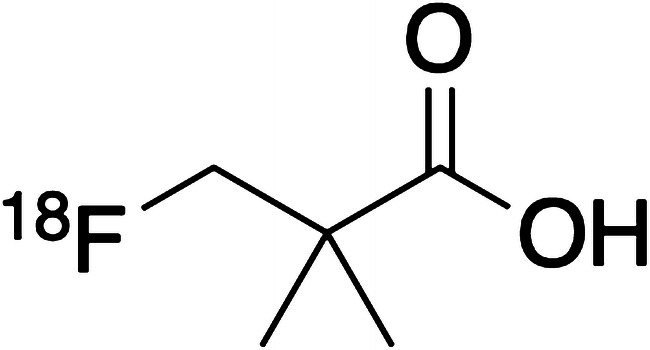
Chemical structure of ^18^F-fluoropivalate (^18^F-FPIA; 3-^18^F-fluoro-2,2-dimethylpropionic acid)

Short chain fatty acids (SCFAs) are rapidly taken up by tumours. For example, in tumour models of malignant glioma, acetate contributes over half of oxidative activity, while glucose contributes only a third [10]. Our group has characterized ^18^F-fluoropivalate (^18^F-FPIA; 3-^18^F-fluoro-2,2-dimethylpropionic acid) to trace SCFA metabolism involving transcellular flux and retention in tumours in non-clinical studies [11](Fig. 1). High tumour uptake of ^18^F-FPIA was seen in murine and human tumour xenografts of the breast, brain, and prostate. This current study presents the first human experience with ^18^F-FPIA, investigating biodistribution, metabolism, and dosimetry, and whether post-prandial effects will for example, necessitate future elaboration of blood carnitine in the use of this radiotracer, analogous to the implementation of the ‘lumped-constant’ for use of ^18^F-FDG [12].

## Materials and methods

### Radiopharmaceutical preparation

The previously reported radiosynthesis of ^18^F-FPIA was adapted to prepare GMP grade radiopharmaceutical using the GE FASTLab™ automated radiosynthesis platform [11, 13]. A detailed description of the automated radiosynthesis is described in the supplementary information (Supplementary material and methods S1). In brief, the precursor methyl 2,2-dimethyl-3-[(4-methylbenzenesulfonyl)oxy] propanoate [1] was radiolabelled by displacement of the tosylate group with dry ^18^F-fluoride to produce intermediate ^18^F-2, the methyl ester of ^18^F-FPIA (Scheme S1). Compound ^18^F2 was hydrolysed under basic conditions to give ^18^F-FPIA which was purified by semi-preparative HPLC using biocompatible solvents (15% EtOH, 85% sodium dihydrogen phosphate buffer, pH 4.5). The fraction containing ^18^F-FPIA was diluted in water and passed through a sterile filter into a sterile vial for clinical use. The identity and purity (chemical and radiochemical purity) of the final product were determined by HPLC; other quality control tests were performed, according to European Pharmacopoeia guidelines (Table S1).

### Subjects

Twenty-four healthy volunteers (12 men, 12 women), were categorised into 2 groups (fed vs fasted). Six men and 6 women were enrolled in the fed group and 6 men and 6 women in the fasted group. Mean age ± SD (59 ± 6.14y); age range, 51–71 y; weight, 76 kg; weight range, 55.3–103.7 kg) were enrolled. In both groups, subjects were asked to fast (water only) from midnight and the fed group were given a light lunch at least 1 h prior to scanning. The fasted group remained fasted until after the scan (approximately 16–18 h). Any subjects taking medications were asked to proceed as normal. Inclusion criteria were age above 50 y, ability to provide written informed consent, and a normal medical history (including physical examination, electrocardiogram, haematology, and biochemistry). Exclusion criteria were pregnancy, lactation, subjects diagnosed with diabetes mellitus, and those with abnormal raised blood lipid levels at baseline as deemed unsuitable for study by clinical investigator. In addition, any chronic illness that would preclude brief discontinuation of medication or musculoskeletal condition that would not allow comfortable performance for the duration of the scan. Subjects receiving any investigational therapy within 14 days or 5 half-lives of a drug prior to the first dose of ^18^F-FPIA injection were also excluded as well as those undergoing monitoring of occupational ionising radiation exposure.

Subjects taking medications (substrates of Cytochrome P450 enzymes-CYP3A4, CYP2C8, and CYP2D6) that may cause any interference were advised to be cautious for at least 7 days and up to 15 days after the last dose of ^18^F-FPIA, due to the potential for alterations in the pharmacologic effects of these medications or an increased risk for serious or life threatening adverse events associated with such medications secondary to the possible inhibition of specific CYP enzymes, by ^18^F-FPIA, as it is metabolised in the liver. Ethical approval for the study was granted by the London-Brent Research Ethics Committee. All volunteers gave written informed consent to participate in the study, according to the Declaration of Helsinki. The administration of radioactivity was approved by the Administration of Radioactive Substances Advisory Committee, U.K.

### Safety, image acquisition, analysis, and Dosimetry

#### Safety

Safety data was obtained during and 24 h after radiotracer administration. Data recorded included vital signs (heart rate, blood pressure, respiratory rate, and body temperature), physical examination, and laboratory parameters (serum biochemistry, haematology, coagulation, and urinalysis). Any adverse events were recorded using the common toxicity criteria (version 5.0: https://ctep.cancer.gov/ protocoldevelopment/electronic_applications/docs/CTCAE_v5_Quick_Reference_5x7.pdf).

#### Image acquisition

Images were acquired on a Biograph 6 TruePoint PET/CT scanner (with TrueV; extended field of view [Siemens]) with 21.6-cm axial and 60.5-cm transaxial fields of view. An attenuation CT scan of each patient was obtained before administration of ^18^F-FPIA, from the vertex to the mid-thigh (CT settings: tube potential, 130 kV; exposure, 15 effective mAs; pitch, 1.5; slice thickness, 5 mm; rotation time, 0.6 s; resulting in an effective dose of 2.5 mSv). This scan was then followed by a multi-bed whole-body PET scanning protocol on 6 occasions within a 4 h period. A break after the fourth PET scan allowed voiding to enhance radiotracer clearance and was followed by a second CT and the last 2 multi-bed whole-body PET scans (Table 1). All emission scans were reconstructed using the ordered-subsets expectation maximization algorithm (3 iterations and 21 subsets) with corrections for dead time, scatter, attenuation, and radioactive decay. Regions of interest (ROIs) for as many of the possible International Commission on Radiological Protection (ICRP), 103 source organs were outlined [14], manually on screen using a circular paint brush of fixed diameter and width on Hermes (Hermes Diagnostics, Stockholm, Sweden) by a single investigator (SD) to avoid any interobserver variation. The bladder was treated separately due to the fact that it increases in volume over the course of scanning. The full bladder volume was outlined in a ROI for each PET scan by a single investigator (NK) and the mean activity concentration and volume recorded to give the total activity in the bladder for each scan.

**Table 1:** Image acquisition protocol

Scan acquisitions	Minute /bed position	No. of bed positions	Vertex to mid-thigh
Attenuation CT*			
PET 1	1	6–7	Vertex to mid-thigh†
PET 2	2	6–7	Vertex to mid-thigh
PET 3	5	6–7	Vertex to mid-thigh
PET 4	5	6–7	Vertex to mid-thigh
Gap			
Attenuation CT 2*			Vertex to mid-thigh
PET 5	7	6–7	Vertex to mid-thigh
PET 6	7	6–7	Vertex to mid-thigh

*CT settings: 130 kV; 15 effective mAs; pitch, 1.5; slice thickness, 5 mm; rotation time, 0.6 s; and effective dose, 2.5 mSv. †Imaging performed caudocranially

#### Blood and urine activity measurements and metabolite analysis

Discrete venous bloods and plasma (at 5, 10, 15, 30, 60, and 120 min post injection (p.i.) were obtained for radioactivity counting and metabolite analysis, as previously described [11]. Urine, ≥ 1 mL, was collected for HPLC measurements of ^18^F-FPIA and metabolites after the 4th and before the 5th and 6th PET scan. Urine (500 μL) was diluted in 500 μL of mobile phase (80:20, sodium phosphate monobasic (0.25 M) + 0.5% tetrabutyl ammonium hydroxide (pH 4.5): methanol), and filtered using 0.22 μm sterile syringe filters; run time 20 min.

#### Non-esterified fatty acids and acylcarnitine measurements

As non-esterified fatty acids (NEFAs) or carnitine levels could potentially modify radiotracer uptake, blood samples were taken for measuring NEFA and carnitine profiles at 3 time-points; at baseline prior to scanning, 60 min p.i. of ^18^F-FPIA, and the end of scan. All samples were centrifuged (1942 g, room temp, 5 min), within 30 min of collection, and stored at −80 °C until transfer to laboratories for analysis.

#### Data analysis, biodistribution and dosimetry

The mean non–decay corrected ^18^F activity concentration was obtained for the source organ ROIs at each whole-body scan time, resulting in time-activity curves (TACs). The activity measured in each bladder ROI was divided by the reference man bladder volume, to give a nominal activity concentration for each point on bladder TAC [15–18].The curves were decay corrected to the mid-point of each whole-body scan to most closely represent the average activity distribution for the scan. The curves were converted to activity per organ using the volume of organs in ICRP 23 reference man [19] and normalized by the injected activity to give the fractional uptake in each organ as a function of time. These TACs were trapezoidally integrated to generate organ residence times (τ); the total number of disintegrations in each source organ per unit injected activity. To account for the activity remaining in the body at the end of the scan protocol, the time-activity curves were extrapolated from the last whole-body scan with the simplification that radioactive decay would be the only significant change. The ED was calculated using firstly the mean residence time over all subjects for each organ and secondly using residence times for individual subjects with OLINDA/EXM v1.1, which uses the organ weighting factors from ICRP 60 [20].

## Results

### Safety

There were no adverse or clinically detectable pharmacologic events during the study or within 24 h after ^18^F-FPIA injection. Two subjects were not able to tolerate the entire scanning duration (4 h). One female fed subject suffered migraine (grade 2) towards the end of the scan (last 42 min) and a second subject expressed fatigue (Grade 1) and was unable to lie down for the full duration of the scan. No significant changes in vital signs or results of laboratory studies or electrocardiograms were observed. Neither of these events were felt to be significant or cause for concern as reviewed by an Independent Data Monitoring Committee appointed for the study.

### Radiopharmaceutical

The automated radiosynthesis of ^18^F-FPIA developed for this study was based on previously described methodology [13], as detailed in the supplemental materials (available at http://jnm.snmjournals.org). A high non–decay-corrected radiochemical yield (RCY) (mean ± SD) of 19.4 ± 1.9% (range, 17.0–22.6%) was obtained and multi-patient doses of ^18^F-FPIA produced (1.5–2.6 GBq). The RCY was theoretically higher (estimated to be 38.9% ± 3.8% n.d.c) as only half of the ^18^F-FPIA crude reaction mixture (10 mL) was purified due to a limitation in the injection loop volume of the semi-preparative HPLC system.^18^F-FPIA was formulated for injection. Radiochemical purity was > 99%, with a pH of 4.68 ± 0.06 (range 4.54–4.76). The mean administered activity was 159.48 MBq (range, 47.31–164.66 MBq). Accurate and reliable measurement of specific activity was not possible by HPLC because of the low ultraviolet absorbance of FPIA, and therefore, high threshold of detectability [11, 13]. As a result, we were unable to quantify amounts of FPIA to the typical low levels seen with other radiotracers. Quality specifications for ^18^F-FPIA were ≤ 12 μg of unknown impurities (including tosic acid) and ≤ 30 μg of non-radioactive FPIA in the total administered dose; these specifications were determined by UV-HPLC.

### Metabolite analysis

**Fig. 2:**
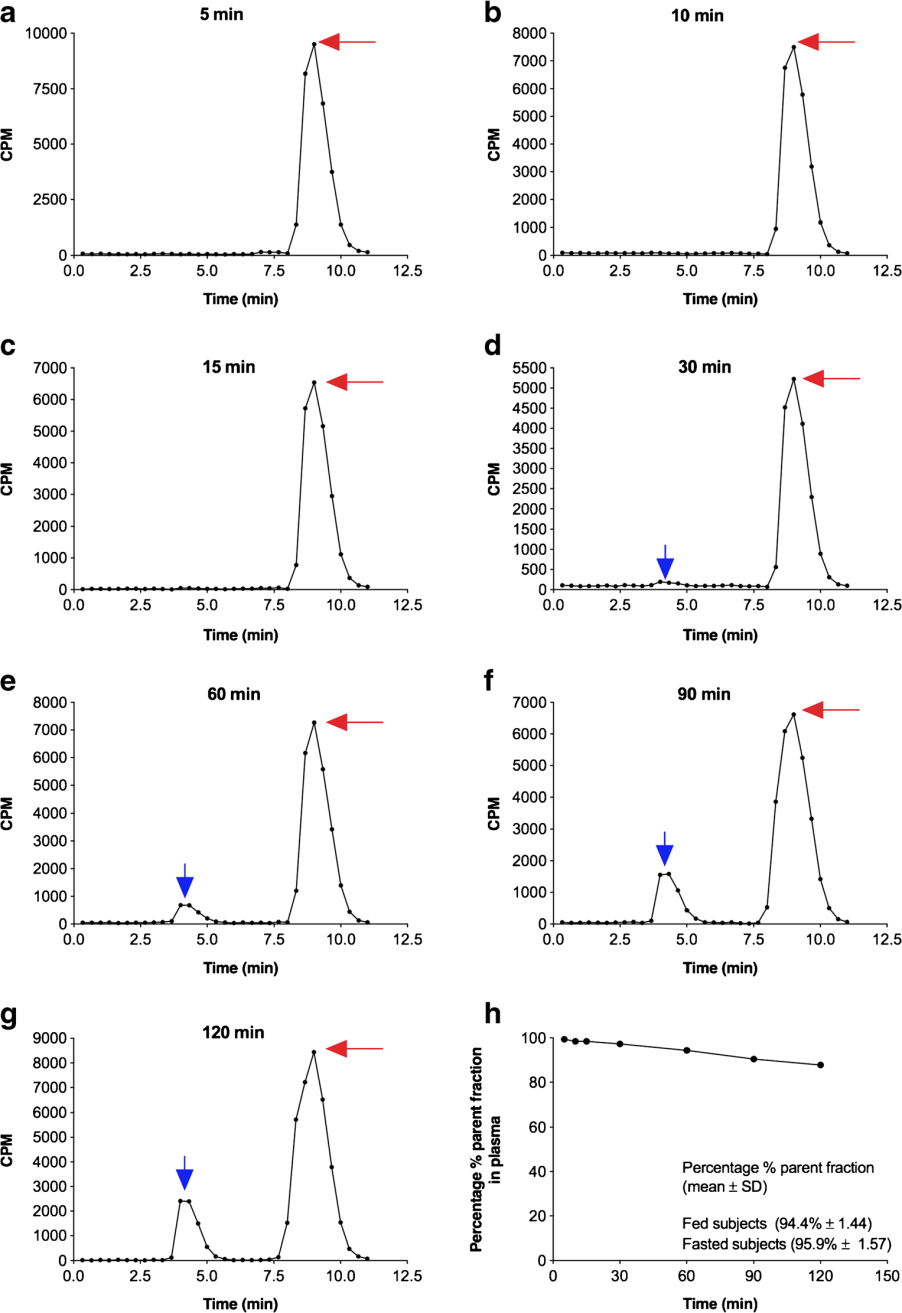
Metabolite analysis of ^18^F-FPIA in a healthy volunteer (fed). (**A–G**) Typical high-performance liquid chromatogram of ^18^F-FPIA in plasma at 5, 10, 15, 30, 60, 90, and 120 min time points. Red arrows indicate parent/unmetabolized ^18^F-FPIA. Scaling of **D–G** adjusted to allow for visualization of metabolite peaks (blue arrow). (**H)** Percentage parent plasma fraction. Over all subjects, 90% parent radiotracer is present after 60 and over 80% at 120 min. In all fed subjects the mean percentage parent tracer after 120 min (94.4% ± 1.44) and in fasted subjects (95.9% ± 1.57). CPM = counts per minute

The metabolism of ^18^F-FPIA was analysed using radio-HPLC (1100 series system; Agilent). Typical HPLC chromatograms of parent tracer ^18^F-FPIA and its metabolites in plasma for subjects are illustrated in Figs. 2 A, B, C, D, E, F, and G. The identity of the plasma metabolites was not elucidated. Over 90% of parent radiotracer was detectable in plasma at 60 min and over 80% at 120 min (Fig. 2H).

The HPLC chromatogram of urinary metabolites taken at approximately 1.15–1.30 h time-point had similar retention times to the plasma metabolites, but of different proportions. The identity of parent ^18^F-FPIA was confirmed by injecting parent compound on the HPLC system. The identity of the major metabolite was tentatively assigned to ^18^F-FPIA-carnitine by comparison to the HPLC retention of analogous pivaloylcarnitine reference (Supplementary Results Fig. 2S).

### Biodistribution

**Fig. 3:**
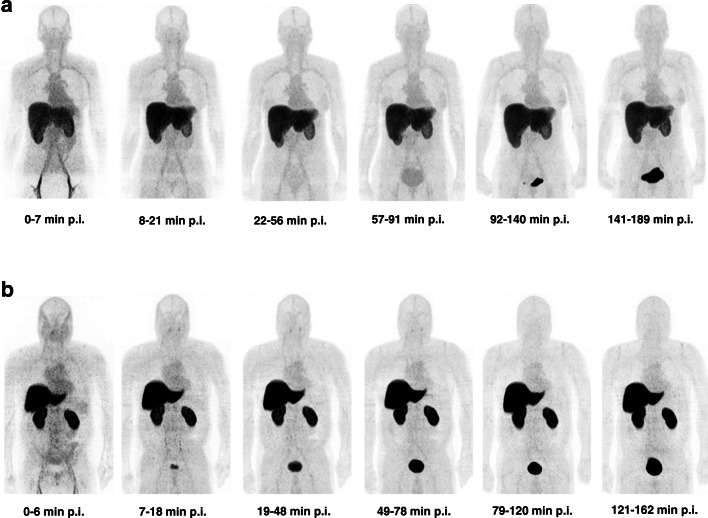
Time course biodistribution of ^18^F-FPIA in female and male healthy subjects. Maximum-intensity-projection images of ^18^F-FPIA in (**A**) female fasted healthy subject and (**B**) male fed healthy subject. p.i. = post injection

**Fig. 4:**
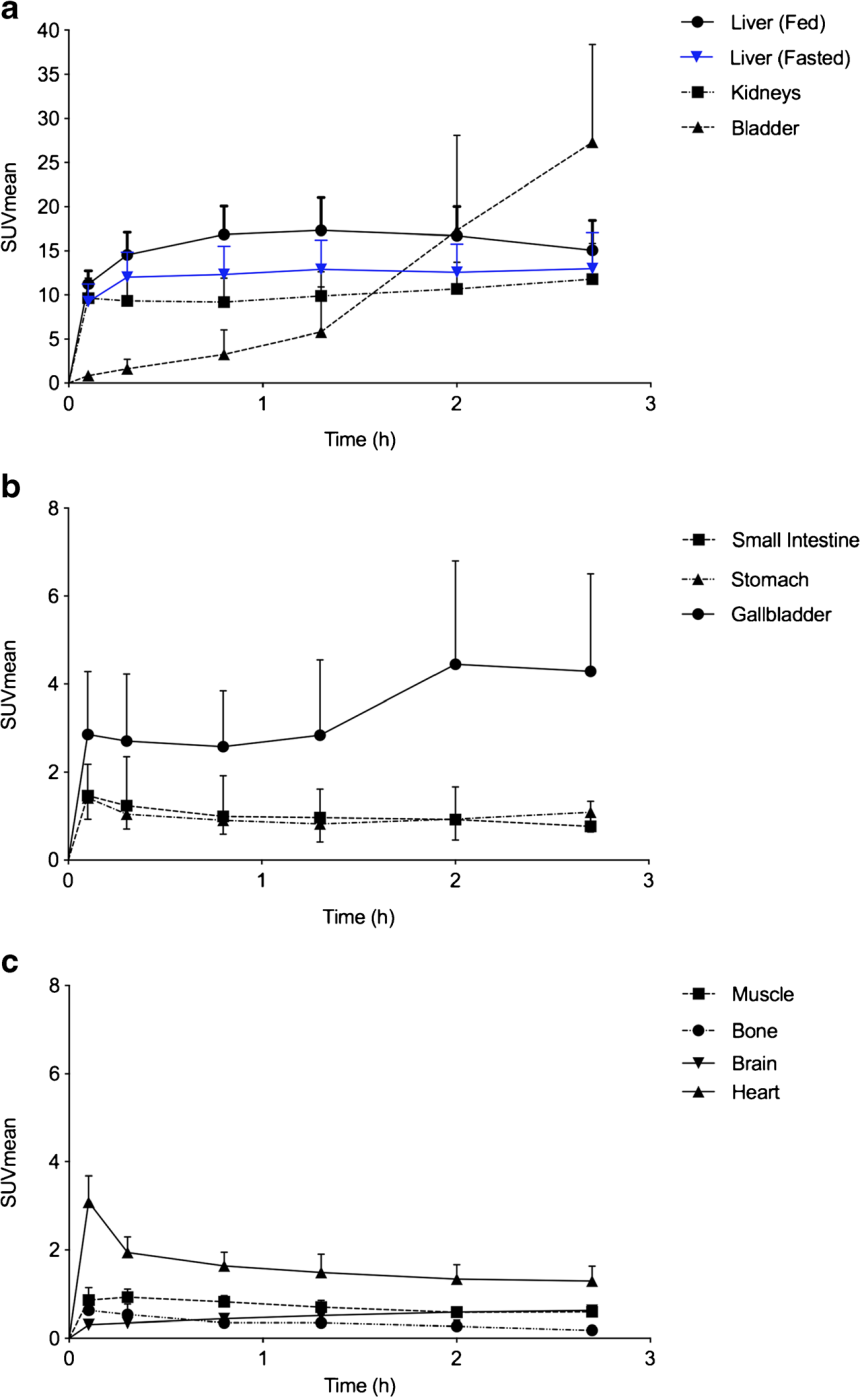
Mean decay-corrected time–activity curves (TAC) for main source organs (**A–C**). Except where indicated, the TACs shown are for fed subjects (*n* = 12 patients). Graph (**A**), also shows the main difference in ^18^F-FPIA uptake between fed and fasted subjects. Fasted subjects showed lower uptake over the course of scanning study

^18^F-FPIA derived radioactivity was visually detectable in the vascular compartment, the liver and kidneys, within the first 6 min of radiotracer injection. No significant differences in organ biodistribution were seen between male and female and fed vs fasted subjects (Fig. 3 and Table 2). Physiological uptake (low-level) was noted in the salivary glands. Tissue time activity curves generated for the main source organs are shown (Fig. 4). Liver uptake (SUV mean) was noted to be higher overall in fed vs unfed subjects (Fig. 4).

**Table 2.** Mean residence times (**τ**) of ^18^F-FPIA for different organs in male (*n* = 12) and female (*n* = 12) subjects and fed (*n* = 12) and fasted (*n* = 12)

**τ** (MBq.h/MBq)								
	Male		Female		Fed		Fasted	
Organ	Mean	SD	Mean	SD	Mean	SD	Mean	SD
Adrenals	4.27E-04	2.89E-04	5.24E-04	3.11E-04	3.50E-04	1.34E-04	6.01E-04	3.68E-04
Brain	1.26E-02	1.57E-03	1.72E-02	4.68E-03	1.41E-02	4.01E-03	1.57E-02	4.18E-03
Breasts			3.72E-03	3.10E-03	1.17E-03	1.84E-03	2.55E-03	3.52E-03
Gallbladder	3.44E-03	1.27E-03	2.71E-03	1.01E-03	3.01E-03	1.16E-03	3.14E-03	1.16E-03
Lower large intestine	7.50E-03	7.68E-03	6.86E-03	2.48E-03	7.67E-03	7.63E-03	6.69E-03	2.57E-03
Small intestine	2.04E-02	7.42E-03	2.44E-02	8.38E-03	1.99E-02	7.61E-03	2.49E-02	7.93E-03
Stomach	8.27E-03	3.10E-03	1.09E-02	4.86E-03	7.46E-03	3.02E-03	1.17E-02	4.30E-03
Upper large intestine	6.98E-03	2.28E-03	9.43E-03	2.11E-03	7.54E-03	2.09E-03	8.88E-03	2.71E-03
Heart	2.24E-02	6.11E-03	2.75E-02	6.36E-03	2.34E-02	5.55E-03	2.65E-02	7.46E-03
Kidneys	4.67E-02	1.07E-02	6.66E-02	2.23E-02	6.06E-02	2.31E-02	5.27E-02	1.56E-02
Liver	4.96E-01	1.24E-01	5.83E-01	2.32E-01	5.98E-01	2.03E-01	4.80E-01	1.57E-01
Lungs	2.48E-02	7.78E-03	3.25E-02	7.45E-03	2.62E-02	4.76E-03	3.10E-02	1.06E-02
Muscle	3.41E-01	5.65E-02	4.44E-01	1.29E-01	3.72E-01	9.29E-02	4.13E-01	1.25E-01
Ovaries			1.01E-04	5.17E-05	4.69E-05	1.38E-05	5.42E-05	3.81E-06
Pancreas	2.36E-03	1.08E-03	4.79E-03	4.10E-03	4.31E-03	4.28E-03	2.84E-03	1.22E-03
Red marrow	1.52E-02	3.84E-03	1.86E-02	4.31E-03	1.51E-02	2.45E-03	1.87E-02	5.14E-03
Cortical bone	2.76E-02	3.28E-02	2.02E-02	6.42E-03	1.79E-02	3.83E-03	2.98E-02	3.25E-02
Spleen	3.94E-03	1.05E-03	4.82E-03	1.42E-03	3.96E-03	1.03E-03	4.80E-03	1.45E-03
Testes	7.35E-04	1.39E-04			3.13E-04	1.09E-04	4.22E-04	1.47E-04
Thyroid	3.56E-04	8.72E-05	5.16E-04	1.54E-04	4.13E-04	1.42E-04	4.60E-04	1.52E-04
Urinary bladder	2.66E-02	8.70E-03	2.33E-02	7.86E-03	2.72E-02	7.98E-03	2.27E-02	8.31E-03
Uterus			2.96E-03	6.57E-04	1.78E-03	5.80E-04	1.18E-03	5.07E-04
Remainder	1.57E+00	2.07E-01	1.34E+00	2.88E-01	1.43E+00	2.99E-01	1.48E+00	2.51E-01

**Fig. 5:**
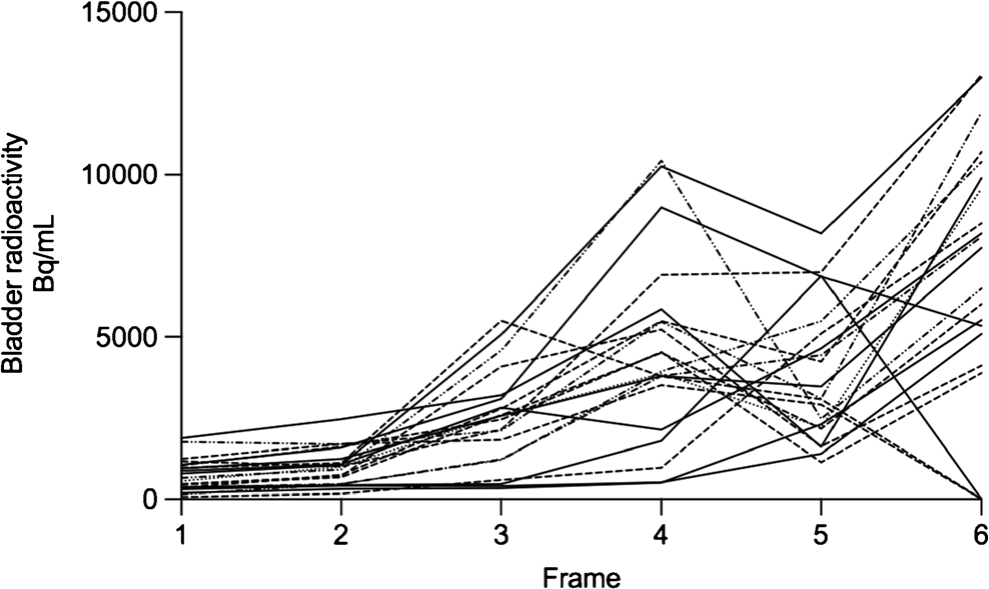
Urinary bladder radioactivity in all subjects. Graph shows variable radioactivity in all subjects within the study. Bladder radioactivity represents the total activity in a standard sized bladder

The mean residence times (τ) in male, female, fed, and fasted subjects are shown (Table 2). Overall, ^18^F-FPIA was eliminated rapidly from most organs leading to relatively short residence times and low/stable organ radioactivity within 60 min of radiotracer injection. Bladder radioactivity was variable in all subjects and could not be fitted to a 3-parameter fit model. Increased tracer radioactivity was seen within the bladder at end of scan, despite voiding after the 4th whole-body PET scan (Fig. 5).

### Dosimetry

The calculated ED using mean organ residence times over all 24 subjects was 0.0154 mSv/MBq (SD ± 0.0010). When using time-activity curves for individual subjects, the calculated ED ranged from 0.0141 to 0.0170 mSv, giving a standard deviation of 0.0010 mSv. The estimated mean absorbed dose to all source organs assuming 2-h bladder voiding scenario in all subjects is shown (Table 3). The organs that received the highest dose (mGy/MBq) in descending order were: liver (0.070 ± 0.023), kidneys (0.043 ± 0.013), gallbladder wall (0.026 ± 0.003), and urinary bladder (0.021 ± 0.004).

**Table 3:** Mean organ-absorbed dose estimates expressed in mGy/MBq for ^18^F-FPIA (*n* = 24)

Mean absorbed dose estimates (mGy/MBq)		
Organ	Mean	SD
Adrenals	1.74E-02	3.35E-03
Brain	4.59E-03	6.29E-04
Breasts	5.18E-03	1.84E-03
Gallbladder	2.60E-02	3.77E-03
Lower large intestine	1.52E-02	3.58E-03
Small intestine	1.74E-02	1.48E-03
Stomach	1.54E-02	1.44E-03
Upper large intestine	1.66E-02	7.98E-04
Heart	1.78E-02	1.23E-03
Kidneys	4.36E-02	1.30E-02
Liver	7.06E-02	2.32E-02
Lungs	1.24E-02	1.53E-03
Muscle	8.89E-03	5.55E-04
Ovaries	9.88E-03	1.14E-03
Pancreas	1.86E-02	6.43E-03
Red marrow	1.07E-02	4.84E-04
Cortical bone	1.53E-02	1.73E-03
Spleen	1.18E-02	1.53E-03
Testes	6.81E-03	9.89E-04
Thyroid	8.92E-03	1.10E-03
Urinary bladder	2.17E-02	4.05E-03
Uterus	1.18E-02	1.65E-03
Total Body	1.24E-02	1.27E-04
Mean ED (mSv/MBq)	1.54E-02	1.00E-03

### Non-esterified fatty acids and acylcarnitine measurements

**Fig. 6:**
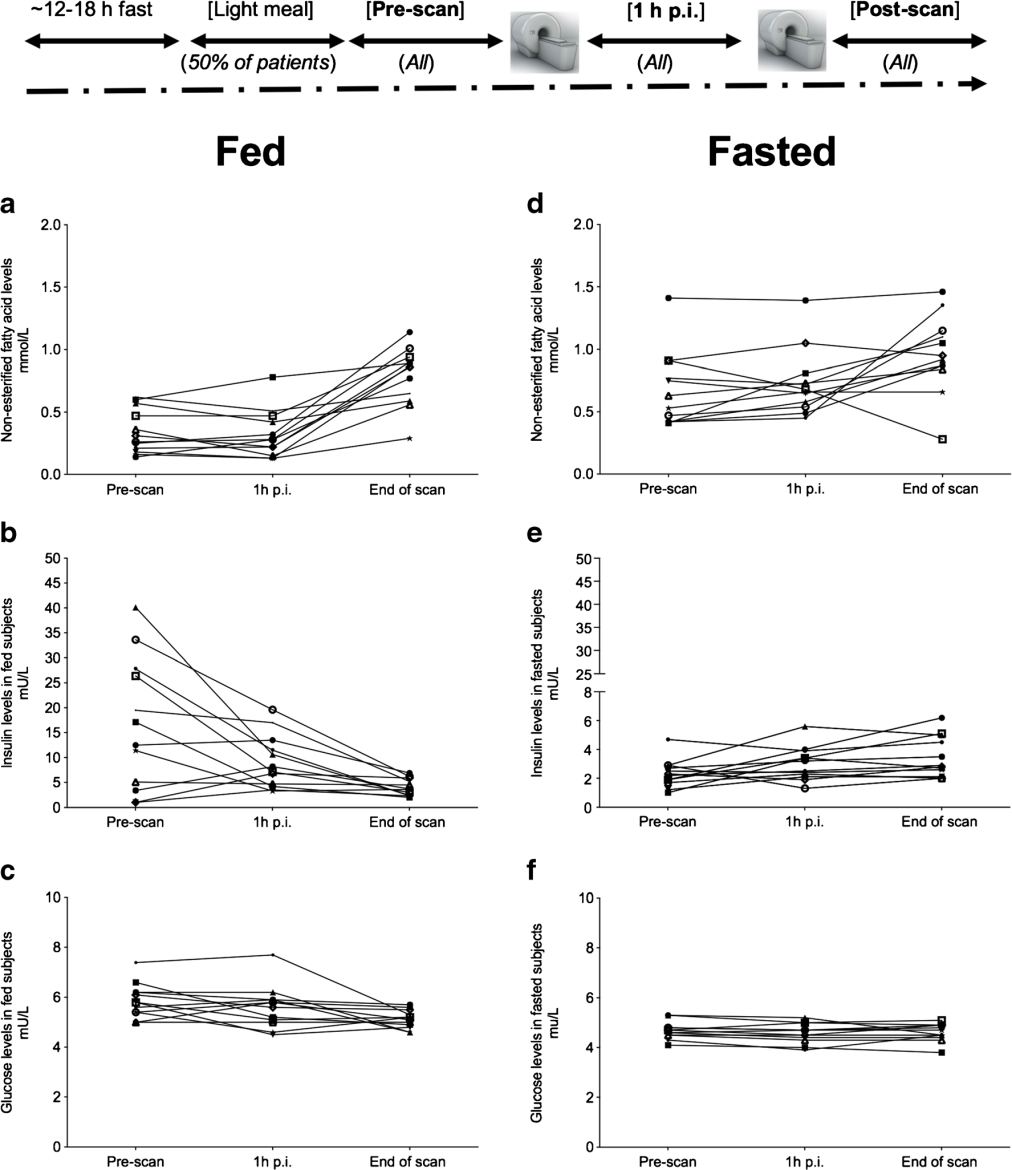
Non-esterified fatty acid, insulin and glucose measurements in blood of fed (**A–C**) and fasted **(D–F**) healthy volunteer subjects

NEFA measurements in fed subjects were low at baseline and showed gradual increase over the course of the scanning study and at end of scan (approx. 2.7–3 h p.i.). Baseline levels were higher in fasted subjects, as expected with a small rise at the end of scan. As expected, high insulin levels were detected at baseline in fed subjects and gradual decline over time with stable glucose levels. In fasted subjects, there were very low levels of insulin secretion with low levels of glucose in blood (Fig. 6).

**Fig. 7:**
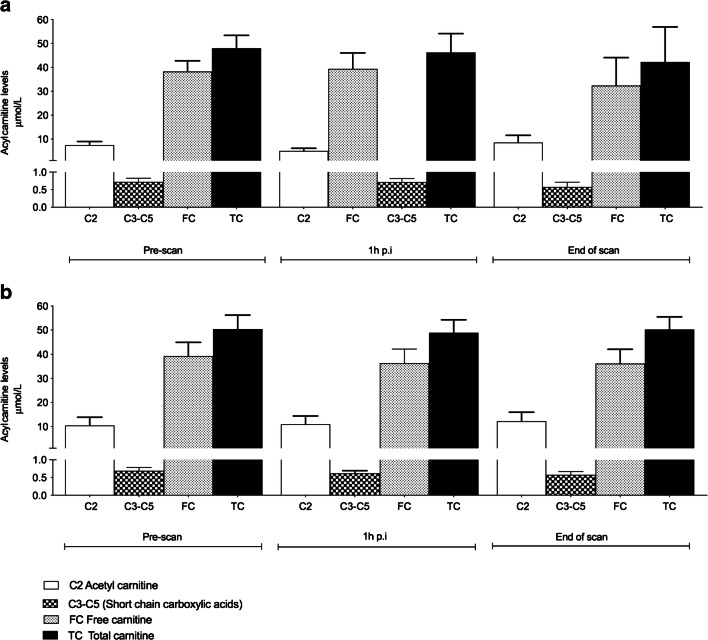
Acyl carnitine measurements in fed and fasted healthy volunteer subjects. Pre-scan (baseline), 1 h p.i. and end of scan blood samples were taken for analysis. Acetyl carnitine (**C2**, short chain fatty acid), (**C3–C5**, sum of short chain carboxylic acids C3, C4, C5; without C2), free carnitine (**FC**) and total carnitine (**TC**) are shown. Overall, levels of C2**,** FC and TC were higher in fasted subjects at baseline, suggesting a flux in the fatty acid oxidation pathway

**Fig. 8:**
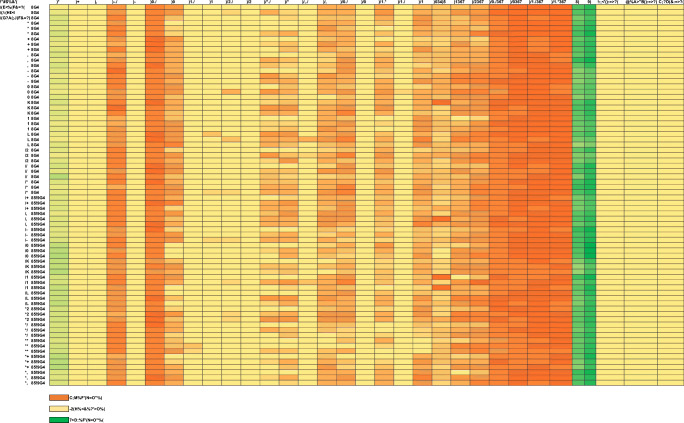
Heat map of Acylcarnitine measurement. Fed and fasted subjects 1–24 are shown (left hand column). For each subject, there are 3 measurements per subject i.e. 1 pre-scan, 1 h p.i. and end of scan. Acetyl carnitine (**C2**), short chain fatty acids (**C3–C5**), medium chain fatty acids (**C6–C8**), Long chain fatty acids (**C10, C12, C14, C16, and C18**), Free carnitine (**FC**), Total carnitine (**TC**)

Carnitine concentration is presented as free (FC) and total (TC); a panel of acylcarnitine short, medium, and long chain fatty acids were found to show minimal differences in fed and fasted groups (Fig. 7 and Fig. 8). Higher baseline acetyl carnitine values (C2, short chain fatty acid), FC and TC were seen in all fasted subjects compared with the fed group. No metabolic disorders or unexpected results were seen.

## Discussion

This first-in-human study shows that GMP-compliant ^18^F-FPIA, a tracer for monitoring SCFA metabolism, is safe, well tolerated, and has suitable dosimetry for use in human PET studies; furthermore, food had little impact on these dosimetry.

The role of fatty acid metabolism, in particular fatty acid oxidation and its contribution to tumour growth and cell survival has come to light over the last few years [4], concomitantly with developments in PET to exploit this phenotype for non-invasive imaging. A number of short and long chain positron-emitting fatty acid analogues including ^11^C-acetate and ^18^F-FAC (2-^18^F-fluoroacetate) have to date been investigated to provide insights into this phenotype in vivo, however, most have limitations. ^11^C-acetate was initially developed for the evaluation of oxidative metabolism in the myocardium [21], with early promise as a tracer in imaging prostate cancer [22]. Other than its short half-life (^11^C, 20.4 min) limiting use to institutions with on-site cyclotron facilities, acetate has multiple fates; it is converted to ^11^C-acetyl-CoA before metabolism to ^11^CO_2_ via the citric acid cycle, and also utilised for de novo fatty acid synthesis. ^18^F-FAC was developed as a mimic of ^11^C-acetate with longer half-life (^18^F, 109.8 min), but is defluorinated in vivo [23]. ^18^F SCFA PET tracers such as ^18^F-FPA (2-^18^F-fluoropropionic acid) and ^18^F-FBA (4-^18^F-fluorobenzoic acid) have also been investigated pre-clinically. However, despite ^18^F-FPA showing promise in prostate cancer imaging, significant uptake was noted in inflammatory lesions and in the bowel [24]. No clinical studies have reported on the use of ^18^F-FPA, and pre-clinical data suggest further modification is required to improve the pharmacokinetics of this tracer before any further development [25]. Regarding long chain fatty acids, ^11^C-palmitate has been developed for use in a number of human experimental cardiovascular studies. Christensen et al. [26] reported the first human biodistribution and dosimetry of ^11^C-palmitate, and despite its suitable dosimetry, this radiotracer showed rapid and complex metabolism including systemic conversion to ^11^CO_2_. Perhaps the most promising long chain fatty acid analogue is 14(R,S)-^18^F-fluoro-6-thia-heptadecanoic acid (^18^F-FTHA) [27, 28]. GMP production of this tracer was achieved in a good radiochemical yield of 13% [29]. Clinical studies are yet to be reported.

In this study, we developed a fully automated GMP production of ^18^F-FPIA for human use, with short synthesis time and we obtained a high non–decay-corrected radiochemical yield (RCY) (mean ± SD) of 19.4 ± 1.9% (range, 17.0–22.6%). Unlike ^18^F-FAC, ^18^F-FPIA showed high metabolic stability with little or no defluorination, indicated by no uptake of radioactivity in bone. The intact parent radiotracer was detectable in plasma by HPLC throughout the study (> 90% parent tracer at 60 min and > 80% at 120 min p.i.), a favourable property that could enable implementation of late imaging protocols. The major metabolite from ^18^F-FPIA systemic biotransformation was tentatively assigned to ^18^F-FPIA-carnitine, which resonates with the metabolism of non-fluorinated pivalate [7–9]. Future studies elucidating the role of this metabolite in OCTN2-mediated cell uptake in tumours will further clarify the mechanism of tumour ^18^F-FPIA-derived ^18^F uptake. The reported renal elimination of the pivalate-carnitine [6] is also resonant with urinary excretion being the dominant route of ^18^F-FPIA elimination. Future studies should explore hydrolysis of the urine sample with base, e.g., KOH to verify conversion back to FPIA. Of note, delayed radiotracer excretion in bladder at late imaging time-points (1.3–2.7 h; PET 4–6; Fig. 5) occurred despite bladder voiding in all subjects after PET4 multi-bed whole body scan, and with all subjects having normal renal function. The rapid radiotracer localisation in the liver, possibly the site of ^18^F-FPIA esterification/turnover, was concurrent with slower bladder elimination.

There were no observable differences in biodistribution between male and female subjects. Low-level physiological uptake was seen in the salivary glands. ^18^F-FPIA uptake and pharmacokinetics were in keeping with pre-clinical studies [11], with rapid distribution to the liver, and gradual elimination over time. As with pre-clinical studies of ^18^F-FPIA, elimination was mainly via urinary excretion [11, 13]. Tracer localisation was primarily noted in the liver, but unlike preclinical studies, localisation in the heart or bowel (small and large) were not observed. Compared to ^18^F-FDG, there was very low uptake within the brain, suggesting potential use of ^18^F-FPIA in future brain tumour imaging efforts. In this study, we investigated the effect of diet on ^18^F-FPIA biodistribution. There were no significant differences in mean SUV uptake in source organs between fed and fasted subjects, with the exception of liver, where mean SUV values were lower overall in fasted subjects. In the fasted (catabolic) state, fatty acid oxidation/ ketogenesis is activated, the liver produces glucose, and lipogenesis is slowed [30]; the converse is true in the fed (anabolic) state.

Dosimetry of ^18^F-FPIA was similar to other ^18^F-based radiotracers. The mean ED was found to be 0.0154 ± 0.0010 mSv/MBq, which is lower than the ED of ^18^F-FDG (0.019 mSv/MBq) [31]. Radiation safety of ^18^F-FPIA was inferred from the organ absorbed dose estimates obtained from our study; all values were within the limits suggested by the US Food and Drug Administration Code of Federal regulation Title 21, Part 361.1.

The levels of blood metabolites were in keeping with normal physiology. In the fasted state, a normal individual would be expected to utilise fatty-acid oxidation pathway resulting in higher baseline levels of NEFA and carnitine (FC and TC). This was observed within our study in all fasted subjects compared with fed subjects. In addition, insulin and glucose levels were as expected low in the fasted state, compared with the fed state where initial insulin levels were high, followed by a transient decline and stable levels of glucose throughout the study. No subject was found to have a metabolic disorder or disorder related to fatty acid oxidation pathway deficiency. From our current knowledge, it would appear that determination of ^18^F-FPIA flux by PET would not require use of a ‘lumped constant’ to describe the relationship between ^18^F-FPIA and carnitine uptake in healthy individuals.

## Conclusion

^18^F-FPIA, a new ^18^F radiolabelled tracer designed for SCFA metabolism, has shown to be safe in humans, with favourable radiation dosimetry and biodistribution including low-level uptake in healthy brain. Furthermore, with the exception of the liver, diet (fed and fasted state) did not impact ^18^F-FPIA biodistribution. This benefit over ^18^F-FDG along with a delayed urinary excretion pattern highlights the potential clinical application for future cancer imaging in both the brain/central nervous system and pelvic malignancies.

## Electronic supplementary material

ESM 1 (DOCX 365 kb)
