# Supplementary material for: Clinical translation of 18F-fluoropivalate – a PET tracer for imaging short-chain fatty acid metabolism: safety, biodistribution, and dosimetry in fed and fasted healthy volunteers
Source: Eur J Nucl Med Mol Imaging. 2020 Mar 2;47(11):2549–61. doi: 10.1007/s00259-020-04724-y (PMC7515955; doi:10.1007/s00259-020-04724-y)
Supplement: Supplementary file 1 — (DOCX 365 kb) [file 259_2020_4724_MOESM1_ESM.docx]

**Supplementary material and methods 1**

**Radiochemistry**

**Scheme S1.** Synthesis of ^18^F-FPIA. *Reaction conditions: a)* ^18^F-fluoride, K_222_, KHCO_3_, DMSO, 120 °C, 25 min; *b)* NaOH, then HCl.

**Automated radiosynthesis of ^18^F-FPIA using the GE FASTlab™ platform.**

An aqueous solution of ^18^F-fluoride in oxygen-18 enriched water (2 – 3 mL) was transferred from the cyclotron target to the hot-cell containing the FASTlab module with a sweep of argon gas. The ^18^F-fluoride was trapped on a Waters Sep-Pak QMA-carbonate light cartridge and eluted into the reactor with a solution (700 uL) containing MeCN (550 uL), H_2_O (150 uL), Kryptofix K_222_ (10.1 mg) and KHCO­_3_ (1.7 mg). The ^18^F-fluoride was dried by evaporation *in vacuo* (120 °C, -1000mBar) and a low flow of nitrogen (200 mBar) for 8 min. To the reactor containing dry ^18^F-fluoride was added **1** (5 - 7 mg) in anhydrous DMSO (500 µL) and heated at 120 °C for 15 min to produce **^18^F-2**. After cooling to 60 °C, a solution of NaOH (1 mL, 2M) was added to the reactor and heated to 50 °C for 15 min to produce ^18^F-FPIA. The reactor was cooled and to the reactor containing crude reaction mixture was added HCl_(aq)_ (1 mL, 2M). The contents of the reactor were transferred to an off-board bottle containing sodium phosphate buffer (17 mL, 58 mM) and phosphoric acid (500 µL, 1M) to achieve a final pH of 2.4 for semi-preparative HPLC purification. The HPLC injection loop (10 mL) was filled and ^18^F-FPIA was purified using an Agilent Eclipse XDB-C18 5µm, 250 × 9.4 mm column with a mobile phase containing 15% EtOH and 85% sodium dihydrogen phosphate buffer (58 mM, pH 4.5) at a flow rate of 4 mL/min. A radioactive peak corresponding to ^18^F-FPIA eluted at ca. 12 min, which was cut for 1 min into a sterile vial and formulated in water for injection (5 mL) to give a final buffered formulation pH 4.5 – 5.0. The final formulation was filtered through a PALL Acrodisc 0.2 μm sterile filter into a final sterile vial (Figure S1).


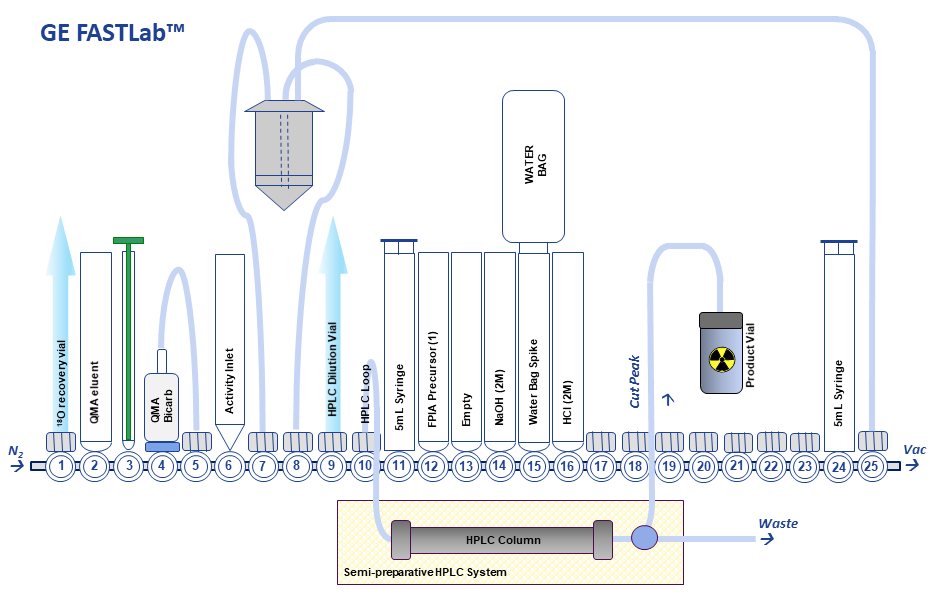


|  | Manifold position |
| --- | --- |
| ^18^O water collection | 1 |
| QMA eluent | 2 |
| QMA-carb SPE | 4-5 |
| ^18^F-inlet | 6 |
| Reaction vessel | 7,8,25 |
| FPIA precursor (**1**) | 12 |
| NaOH | 14 |
| HCl | 16 |
| Water | 15 |
| HPLC dilution vial | 9 |
| HPLC loop | 10 |
| Product vial | Off cassette |
|  |  |

**Figure S1.** Schematic representations of the GE FASTLab™ cassette with HPLC purification.

**Table S1.** ^18^F-FPIA product description

|  | Criteria |
| --- | --- |
| Appearance  pH  Chemical purity^a^  Kryptofix 222  Stable FPIA  FPIA precursor (**1**)  Hydroxypivalic acid  Unknown impurities  Residual solvents  Acetonitrile  DMSO  Ethanol  Radionuclide identity  Radiochemical purity  Radiochemical stability  Sterility  Endotoxin^a^ | Clear, colourless, practically free from particles  4.5 – 7.0  ≤ 2.2 mg  ≤ 30 μg  ≤ 12 μg  ≤ 4.1 mg  ≤ 50 mg  3 – 10%  105 – 115 min  ≥ 95 %  ≥ 95 %^b^  Sterile  ≤175 EU |

^a^ in total administered dose
^b^ at end of shelf-life

**Supplementary Results 1**

#

| **A**   | |
| --- | --- |
| **B**  **^18^F-FPIA Parent**  **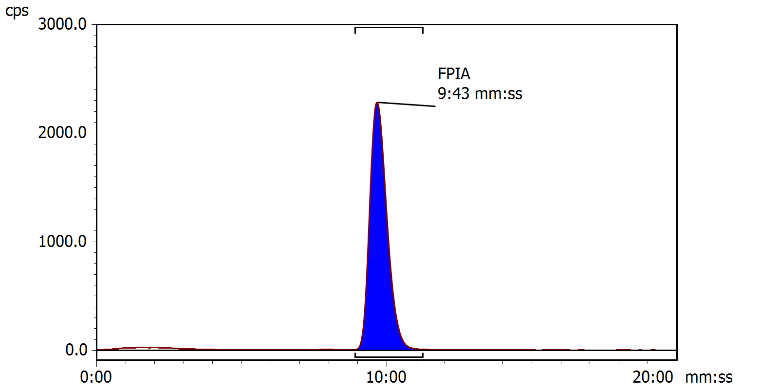** | **C**  **Pivaloylcarnitine**  **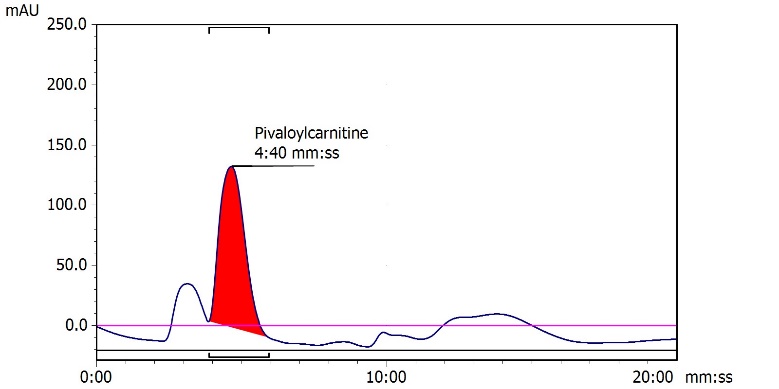** |
| **D**  **Radioactive metabolites in urine**  **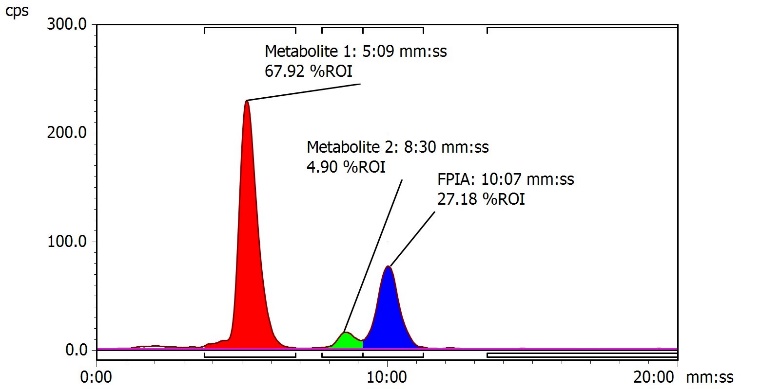** |  |

**Fig S2. A)** Putative mechanism for the *in vivo* synthesis of ^18^F-FPIA-carnitine based on a published mechanism for the *in vivo* metabolic fate of pivalic acid *via* activation by coenzyme A (11). **B)** Radio-HPLC chromatogram showing ^18^F-FPIA parent radiotracer, t_R_ = 09:43 min:sec. **C)** UV-HPLC chromatogram of pivaloylcarnitine obtained from a commercial supplier, t_R_ = 04:40 min:sec, λ = 210 nm. **D)** Representative radio-HPLC chromatogram showing a main radioactive metabolite (t_R_ = 05:09 min:sec) in urine post PET scan. There is a 20 – 30 sec delay between the UV detector and γ-detector on the HPLC system used.
